# Stridor caused by endobronchial lymphoma in a middle‐aged woman

**DOI:** 10.1002/rcr2.1335

**Published:** 2024-03-25

**Authors:** Chih‐hsi Pan, Chien‐wen Chen

**Affiliations:** ^1^ Division of Pulmonary and Critical Care Medicine, Department of Internal Medicine Tri Service General Hospital, National Defense Medical Center Taipei City Taiwan

**Keywords:** diffuse large B‐cell lymphoma, endobronchial lesion, stridor

## Abstract

Diffuse large B‐cell lymphoma, primarily nodal in nature, can present with rare endobronchial involvement, underscoring the importance of considering it in the differential diagnoses of endobronchial lesions.

## Graphical abstract

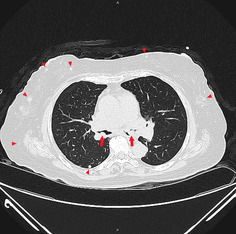

## CLINICAL IMAGE

**FIGURE 1:**
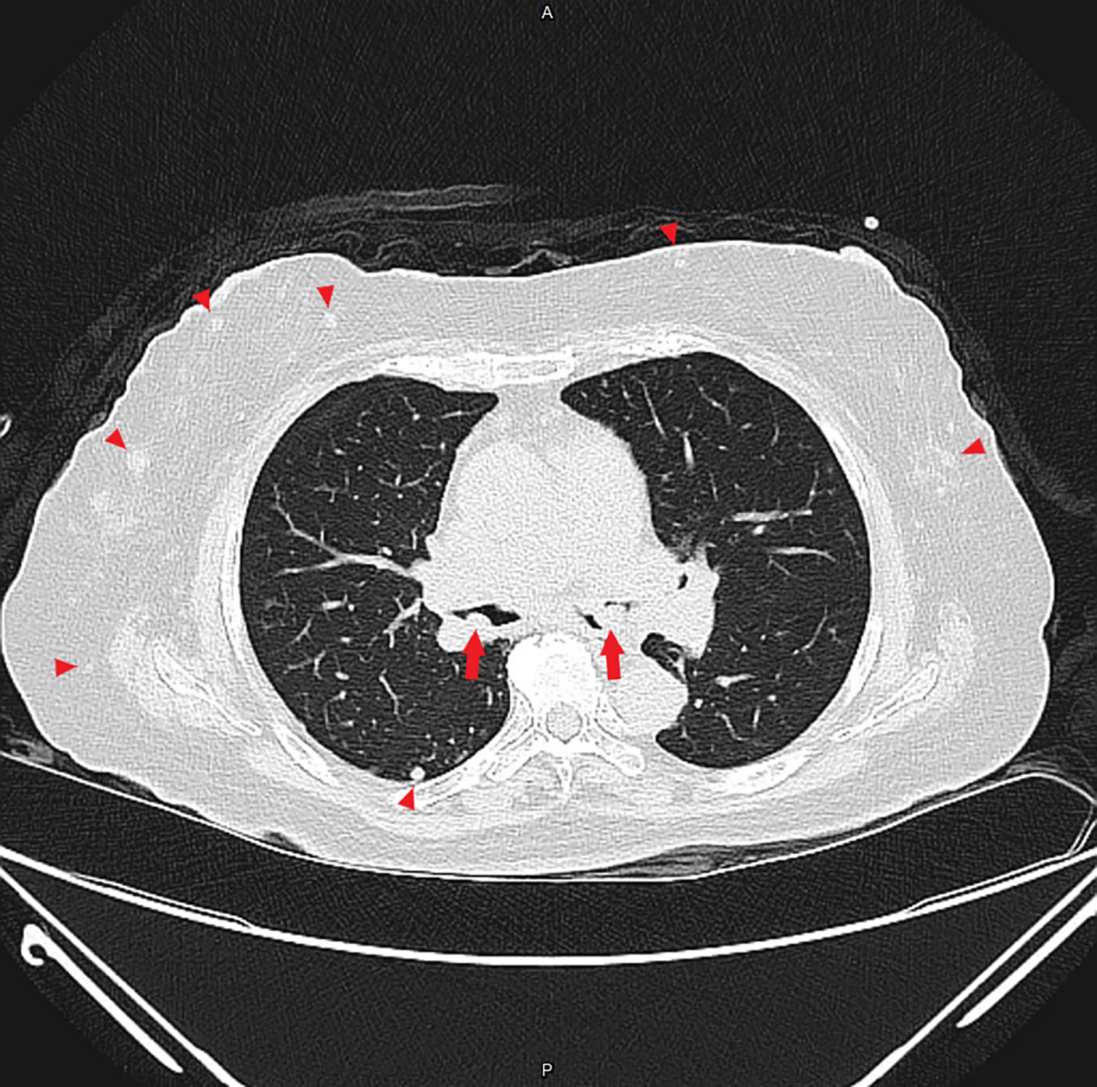
Non‐contrast chest CT showed multiple nodular lesions (arrowheads) in the subcutaneous tissue and lungs. Additionally, multiple endobronchial lesions (arrows) were also observed.

**FIGURE 2:**
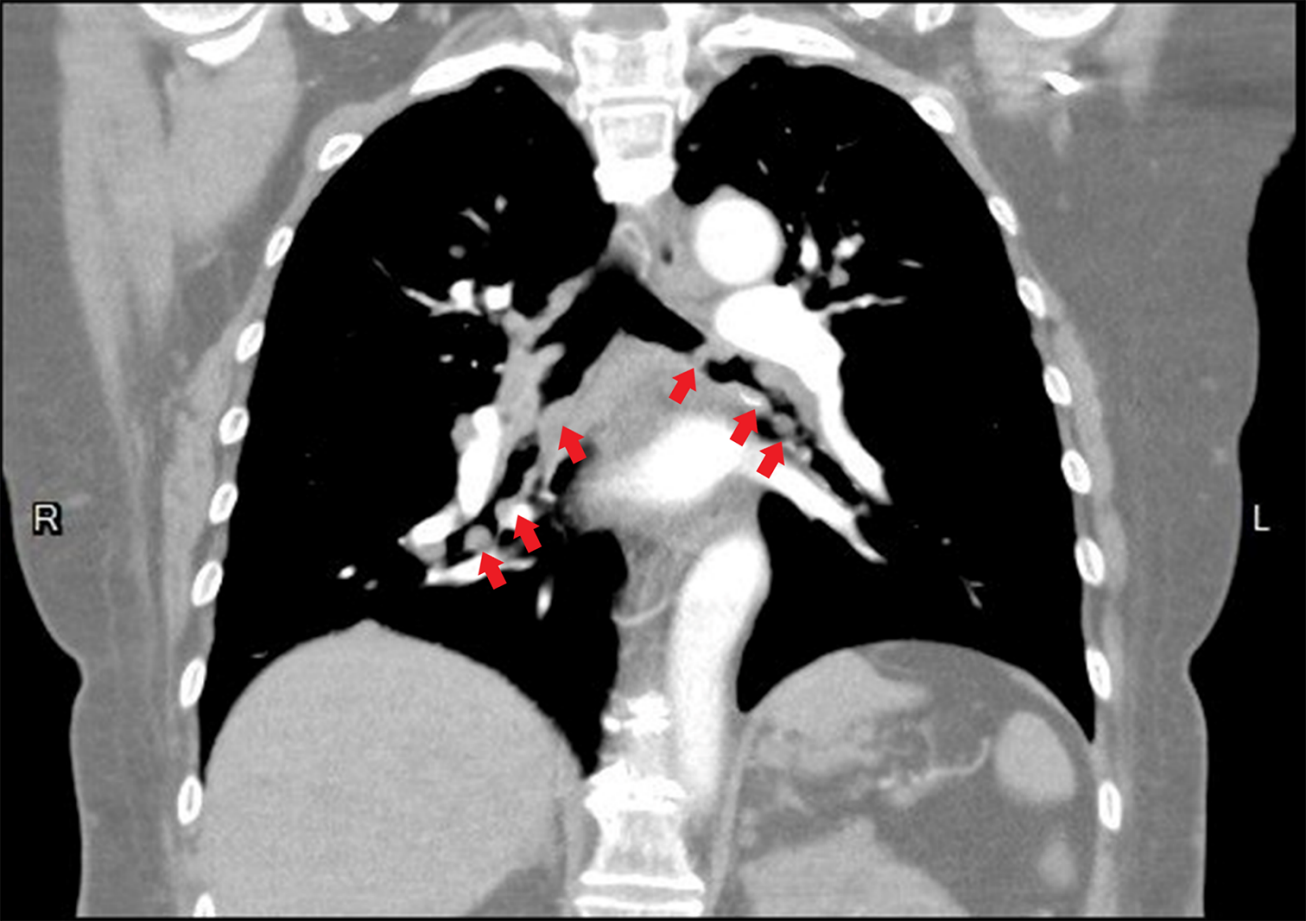
Contrast chest CT showed multiple tracheobronchial nodules in primary and secondary bronchus (Coronal view).

The 63‐year‐old female, without a history of systemic diseases, presented to our emergency department with complaints of shortness of breath and low‐grade fever persisting for 2 days, without any accompanying night sweats or weight loss. Upon examination, bilateral stridor and wheezing were observed. A chest computed tomography (CT) (Figure 1) image revealed multiple subcutaneous nodules in the chest wall, numerous pulmonary nodules predominantly in the upper zones, and multiple tracheobronchial nodules in primary and secondary bronchus (Figure 2) with suspected posterior membrane involvement and luminal narrowing. These findings raised suspicion of neurofibromatosis, amyloidosis, or granulomatosis with polyangiitis.

**FIGURE 3:**
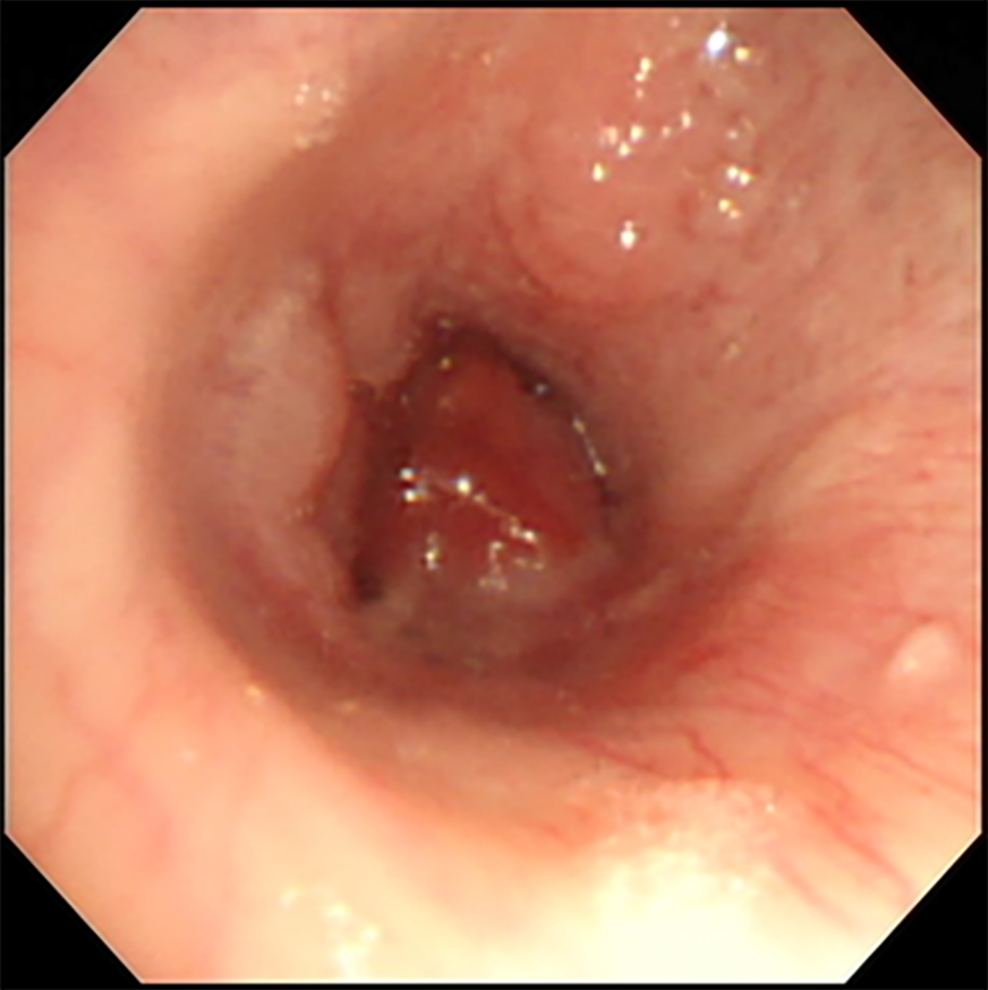
Polypoid endobronchial lesion occluding right lower lobe.

**FIGURE 4:**
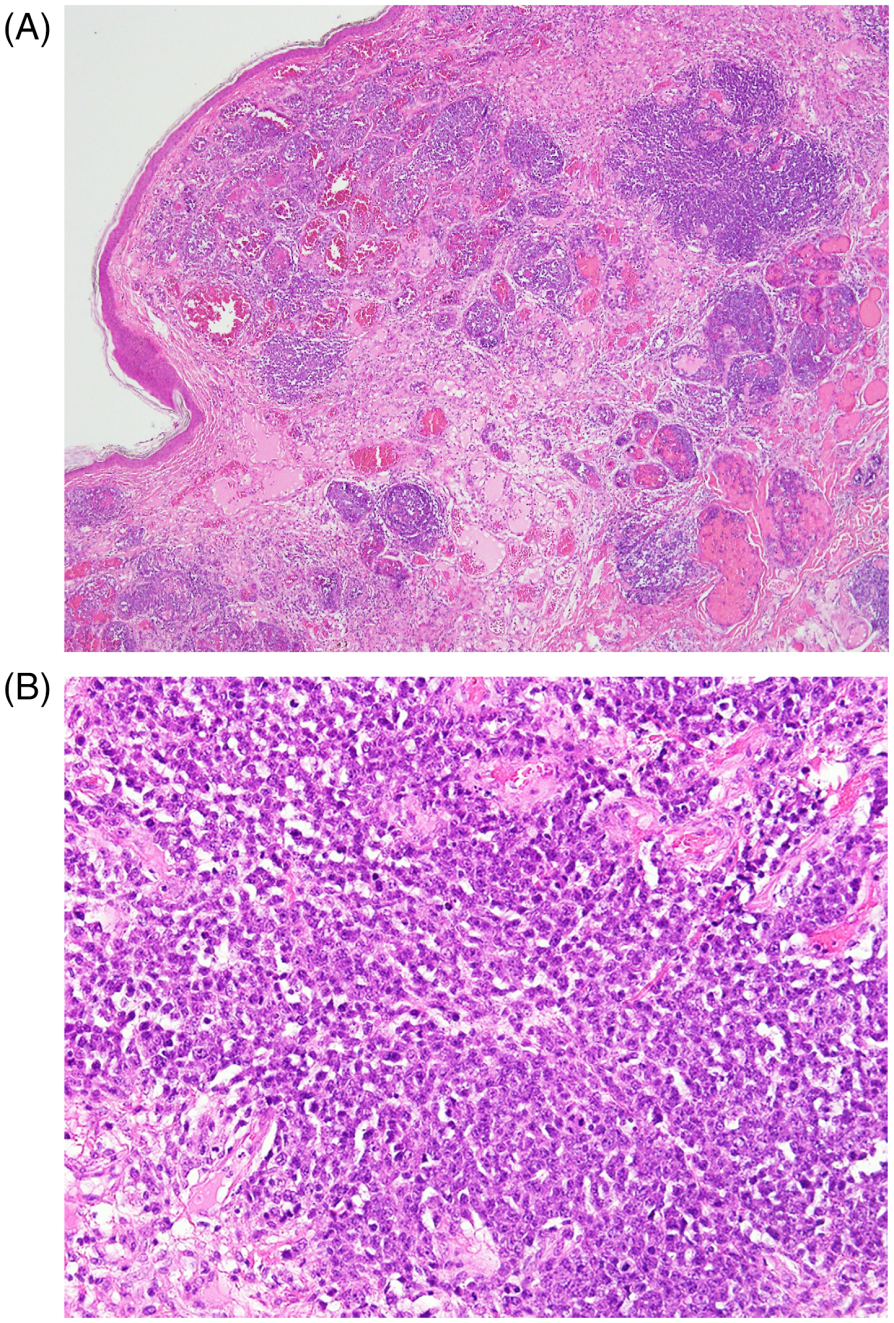
(A, B) The skin biopsy specimen revealed a histological appearance characterized by monotonous enlarged lymphoid tumour cells with hyperchromatic nuclei arranged in a sheet‐like pattern.

**FIGURE 5:**
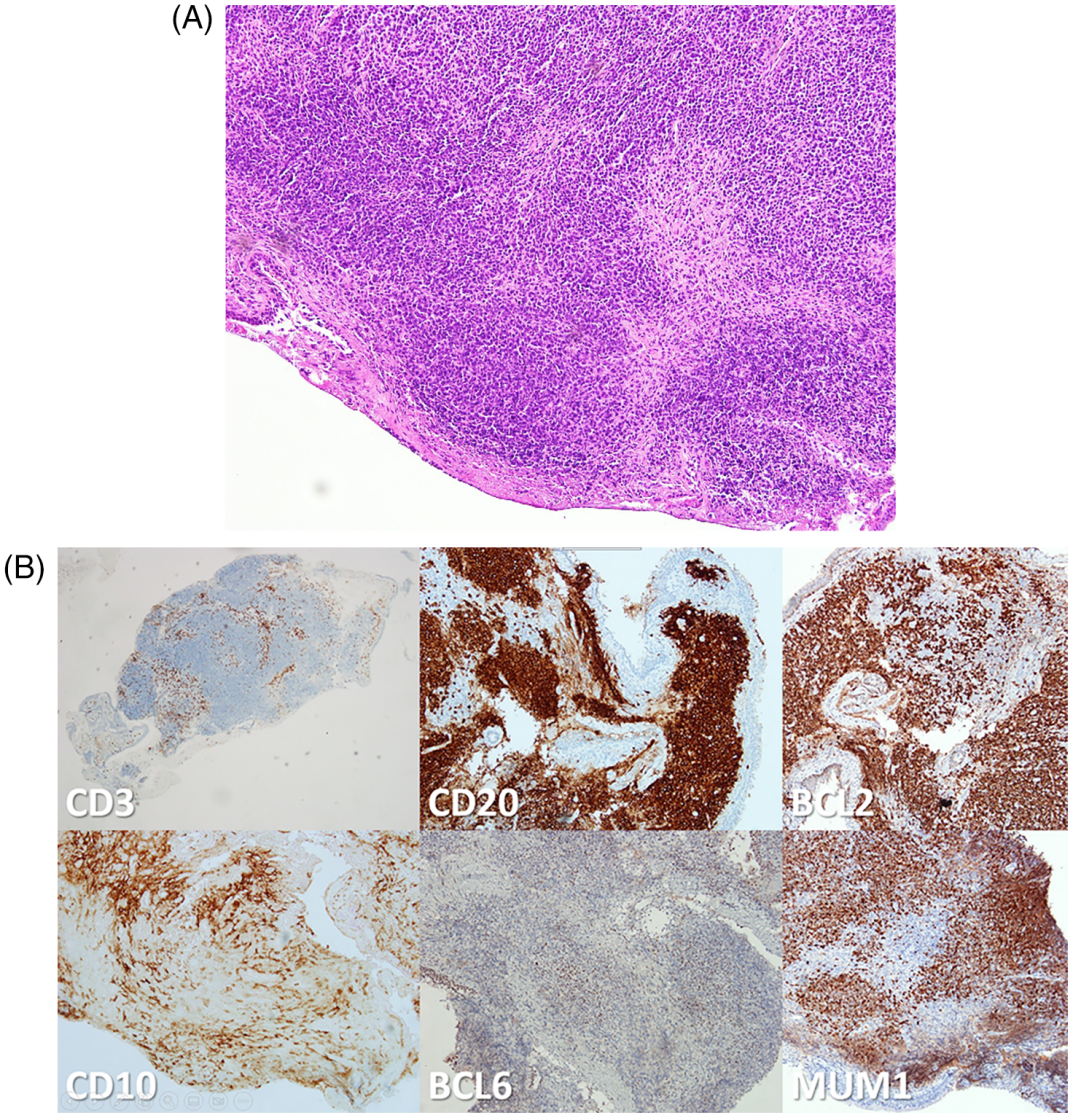
(A) The lesions biopsied from within the bronchus also exhibited characteristics identical to those of the skin biopsy specimen. (B) Immunostaining showed positive finding of CD10 CD20, BCL2 and MUM1.

Initially, a skin biopsy was performed for safety reasons. After multidisciplinary discussion, biopsy of the tracheal lesion was also obtained via bronchoscopy tracheal (Figure 3). Histopathological analysis (Figure 4) revealed monotonous enlarged lymphoid tumour cells with hyperchromatic nuclei arranged in a sheet‐like pattern. Immunostaining (Figure 5A,B) showed positive finding of CD10 CD20, BCL2 and MUM1 confirmed the final diagnosis diffuse large B‐Cell lymphoma. In conclusion, diffuse large B‐cell lymphoma, primarily nodal in nature, can present with rare endobronchial involvement, underscoring the importance of considering it in the differential diagnoses of endobronchial lesions.

## AUTHOR CONTRIBUTIONS

Chih‐hsi Pan was involved in investigation, writing original draft, writing review and editing and final approval of the manuscript. Chien‐wen Chen was involved in the supervision.

## CONFLICT OF INTEREST STATEMENT

None declared.

## ETHICS STATEMENT

The authors declare that appropriate written informed consent was obtained for the publication of this manuscript and accompanying images.

## Data Availability

Data sharing is not applicable to this article as no new data were created or analyzed in this study.

